# Evaluating the Effect of Three Water Management Techniques on Tomato Crop

**DOI:** 10.1371/journal.pone.0129796

**Published:** 2015-06-09

**Authors:** Mohammad Nabil Elnesr, Abdurrahman Ali Alazba, Assem Ibrahim Zein El-Abedein, Mahmoud Maher El-Adl

**Affiliations:** Alamoudi Chair for Water Research, King Saud University, Riyadh, Saudi Arabia; Northwest A&F University, CHINA

## Abstract

The effects of three water management techniques were evaluated on subsurface drip irrigated tomatoes. The three techniques were the intermittent flow (3 pulses), the dual-lateral drip system (two lateral lines per row, at 15 and 25cm below soil surface), and the physical barrier (buried at 30 cm below soil surface). Field experiments were established for two successive seasons. Water movement in soil was monitored using continuously logging capacitance probes up to 60 cm depth. The results showed that the dual lateral technique positively increased the yield up to 50%, water use efficiency up to 54%, while the intermittent application improved some of the quality measures (fruit size, TSS, and Vitamin C), not the quantity of the yield that decreased in one season, and not affected in the other. The physical barrier has no significant effect on any of the important growth measures. The soil water patterns showed that the dual lateral method lead to uniform wetting pattern with depth up to 45 cm, the physical barrier appeared to increase lateral and upward water movement, while the intermittent application kept the wetting pattern at higher moisture level for longer time. The cost analysis showed also that the economic treatments were the dual lateral followed by the intermittent technique, while the physical barrier is not economical. The study recommends researching the effect of the dual lateral method on the root growth and performance. The intermittent application may be recommended to improve tomato quality but not quantity. The physical barrier is not recommended unless in high permeable soils.

## Introduction

While the presence of water in the root zone is vital for plants, the soil wetting pattern (WP) has a significant effect on the crop growth [1]. The WP depends on two major factors: the soil properties (texture, hydraulic conductivity, etc.) [2–4] and the application scheme (application method, position, rate, and frequency).[4–7].

To conserve water within the root zone, some researchers suggested placing an impermeable barrier (made of polyethylene or foil) below the dripper lines to limit the downward movement of water [8–10]. The application of such technique in soils with high infiltration rates increased the crop yields significantly [11,12]. On the other hand, the installation of such physical barrier is tiring and costly, it may causes root rot or shallow root diseases, in addition to the potential hazards of salts accumulation and other chemical toxicity problems [13].

A different concept that was introduced by Ismail et al. [14] that involves burying two dripper lines, one below the other, where the two lines emit the same amount of water that is used for the single dripper line. This dual-lateral method is based on an assumption that due to the pressure head gradient, water moves faster into the dry soil than into the moist soil, and thus, when the secondary drip line moistens the soil below the primary drip line, it drives water from the upper drip line to redistribute upward and laterally, rather than moving downward. Hence, they called this technique “a hydraulic barrier”. This technique showed up to 48% increase in the Jerusalem artichokes yield, while it showed varying results for tomato depending on the distance between the two laterals [14].

Another important technique is the drip intermittent application. Depending on the good results of the intermittent application of flood irrigation to improve water uniformity and increase crop yield [15], several investigators used the same concept for drip irrigation, reporting that the intermittent application limits emitters clogging [16], increases yield and water use efficiency [17–20], decrease chemicals and fertilizers usage [21], saves water and reduce deep percolation [22,23].

In this work, we aimed to evaluate the effect of the three water management techniques; the physical barrier (P), the dual-lateral drip system (H), and the intermittent flow (S) on the wetting pattern, and on tomatoes (*Solanum lycopersicum* L.) yield and water use efficiency.

## Material and Methods

### 2.1 Region and field conditions

**Fig 1:**
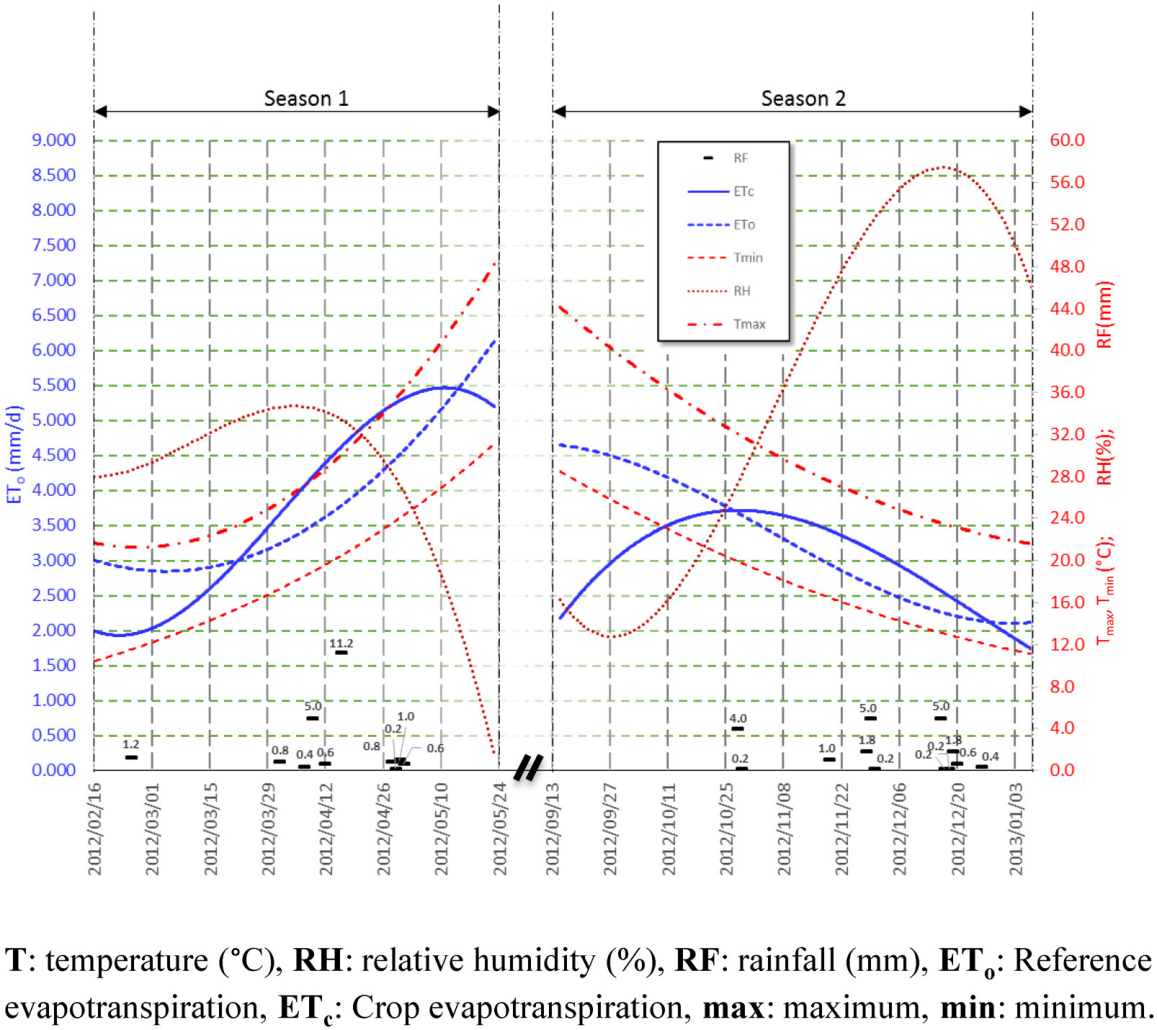
Climatic trends for the two studied seasons.

The field study was carried out in Riyadh, Saudi Arabia, in the Educational farm of the King Saud University, 24°44'12.66"N and 46°37'13.32"E, no need of any special permission to carry out the study, the field experiment was carried out on plant only, not vertebrates, and it did not involve endangered or protected species. The field’s area was 32 × 19 m^2^. The climate of the region is arid [24] with very little precipitation through the year, except some flash rains in March and April. In summer months, the weather is extremely hot, while in winter, it is mild with some few winds and sand storms. The experiments were repeated for two open-field seasons, 2/2012 (hot season), and 9/2012 (cold season). The trends of the maximum and minimum temperatures, relative humidity, rainfall, and reference and crop evapotranspiration values are illustrated in Fig 1.

### 2.2 Soil properties

The soil of this field was sandy loam up to 60 cm depth. At the beginning of the experiment, we took soil samples at 0–20, 20–40, 40–60 cm depths, the samples were air-dried and gently crushed and sieved through a 2 mm sieve, and stored for chemical and physical analysis. Particle size distributions were achieved according to Gee and Bauder [25]. Soil pH and EC values were determined in soil paste using a pH-meter according to Thomas [26] and EC-meter according to Rhodes [27]. Calcium carbonate was determined using a calcimeter according to Loppert and Suarez [28]. The concentration of soluble Cations and anions (Ca^2+^, Mg^2+^, Na^+^, K^+^, CO_3_^2-^, HCO_3_^-^, Cl^-^ and SO_4_^2-^) was determined according to the methods described in Sparks et al. [29]. Finally, the soil organic matter content was determined according to Nelson and Sommers [30]. The soil properties are listed in Table 1.

**Table 1:** Soil Properties of the study field.

**Depth**	**Sand**	**Silt**	**Clay**	**pH**	**EC**	**PWP**	**FC**	**Sat.**	**CaCO_3_**	**Texture class**
**[cm]**	**[%]**	**[%]**	**[%]**		**[dSm^-1^]**	**[cm^3^.cm^-3^]**	**[cm^3^.cm^-3^]**	**[cm^3^.cm^-3^]**	**[%]**	
0–20	68.99	17.60	13.42	7.57	3.64	0.059	0.191	0.357	20.00	Sandy Loam
20–40	69.30	17.60	13.11	7.41	2.93	0.061	0.202	0.359	20.72	Sandy Loam
40–60	79.19	10.22	10.60	7.49	2.29	0.058	0.175	0.365	14.42	Sandy Loam
**Depth**	**Cations [meq/l]**				**Anions [meq/l]**				**OM**	
**[cm]**	**Ca^2+^**	**Mg^2+^**	**Na^+^**	**K^+^**	**HCO_3_**	**CO_3_^=^**	**Cl^-^**	**SO_4_^=^**	**[%]**	
0–20	12.50	8.05	13.33	0.49	2.70	0.00	3.25	31.00	0.14	
20–40	10.00	8.75	8.99	0.54	3.70	0.00	1.75	22.40	0.07	
40–60	6.75	6.75	7.88	0.37	3.05	0.00	1.08	17.40	0.00	

**EC**: Electrical conductivity, **PWP**: Permanent wilting point, **FC**: Field Capacity, **Sat.**: Saturated water content, **OM**: Organic matter.

### 2.3 Experimental design

The three studied techniques were applied each in two levels; applied {1} and not applied {0}. The physical barrier’s levels were P_1_ and P_0_ for applied and not applied respectively. Similarly, the dual-lateral technique is denoted H_1_ and H_0_ respectively, and the intermittent application (surge drip) is denoted S_1_ and S_0_ respectively. The selected experimental design was split split-plot factorial design in 2^3^ (8) treatments including interactions, each treatment was applied on nine row crops (replicates). The statistical design details are in section 2.8.

### 2.4 Tomatoes planting

Seeds of commercial tomato variety Kilele F1 were sown in seedling trays (Jiffy-7 peat pellets, Moerdijk, The Netherlands). The seeds were grown in fiberglass greenhouse under controlled conditions at temperatures of 25±1°C/day and 20±1°C/night. After four weeks of seed sowing, seedlings of uniform size having five true leaves were transplanted outdoor (at the open field) into rows of 7 m length and 0.85 m width. The distance between plants was 50 cm. Fertilization and other cultural practices were applied as commonly recommended in commercial tomato production [31].

### 2.5 Irrigation design and scheduling

For all plots in the subsurface drip network, we applied the following parameters: the main lateral line is buried 15 cm below soil surface, with 4L/h built-in emitters ≅33 cm apart (3 emitters/m) (Rhein dripperline, pressure compensating, clogging resistance, Saudi drip, K.S.A). For the dual lateral plots (H_1_), an additional lateral line is buried 10 cm below the main lateral line (25cm below soil surface); the scheduled amount of water is divided equally between the two laterals. For the continuous flow treatments (S_0_), all the scheduled irrigation was applied at once. The scheduled water for intermittent flow treatments (S_1_) was divided into equal amounts according to the selected pulse rate (3 pulses), where the OFF duration is three times as the ON duration (a numerical example is at the end of this section).

For the treatments with physical barrier (P_1_), the barrier was placed 30 below the soil surface. The form of the physical barrier is a semicircular PVC tube, 110 mm width; this is narrower than the physical barrier of Ismail et al. [14] (500 mm width), but it is wider than the size of Brown et al. [10] which was less than 57 mm L shaped strip. The amount of water applied to all plots were adjusted to be the same, the irrigation process was scheduled by calculating the crop evapotranspiration by the method of FAO 56 [32], according to the weather station nearby in the same farm. The values of the crop coefficients were taken from FAO 56 as 0.6, 1.15, and 0.9 for the initial, medium, and end growing stages. According to the numbers of emitters in each plot, the desired amount of water is converted to the equivalent operation time and then programed to automatic controllers, (Rainbird ESP Modular Controller, Rainbird, USA).

#### 2.5.1 A numerical example of the timing calculation

Assume the desired amount of irrigation water is 9.6 mm.d^-1^ for the control treatment. The required time to apply this amount should be calculated depending on soil properties, emitters properties, number of emitters, and area of the plot [33] by Eq (1).

$$T=\frac{A\cdot d_{g}}{Q}$$ (1)

where *T*: irrigation time, T; *A*: wetted area (normally considered 50% of the drip irrigated area), L^2^; *d*_*g*_: gross irrigation depth, L; *Q*: applied water discharge, L^3^T^-1^). For the irrigated plot if *A* = 80 m^2^, *Q* = 1.92 m^3^ h^-1^, and *d*_*g*_ = 9.6 mm.d^-1^, then *T* = 24 minutes. This amount of time will be applied to the control treatment (S_0_H_0_) and the system will work continuously for 24 minutes. For the plots with dual lateral, S_0_H_1_, the amount of discharge (*Q*) is doubled, and hence the irrigation time is halved, i.e., the plot will work for 12 minutes continuously. For the pulsed treatments with single lateral, S_1_H_0_, the required operation time will be 24 minutes but it will be divided according to the following timing profile: 8-24-8-24-8 where the eights are the ON times and the 24s are the OFF times. For the pulsed treatments with dual lateral, S_1_H_0_, the operation profile would be as follows 4-12-4-12-4, for total 4×3 = 12 minutes total of ON times and 24 minutes of OFF times. We also apply the same amount of water to the S_0_H_1_ plots, since the H_1_ plots has double the number of emitters than the H_0_ plots, then the required time will be half the initial time, i.e. 12 minutes.

### 2.6 Water measures

**Fig 2:**
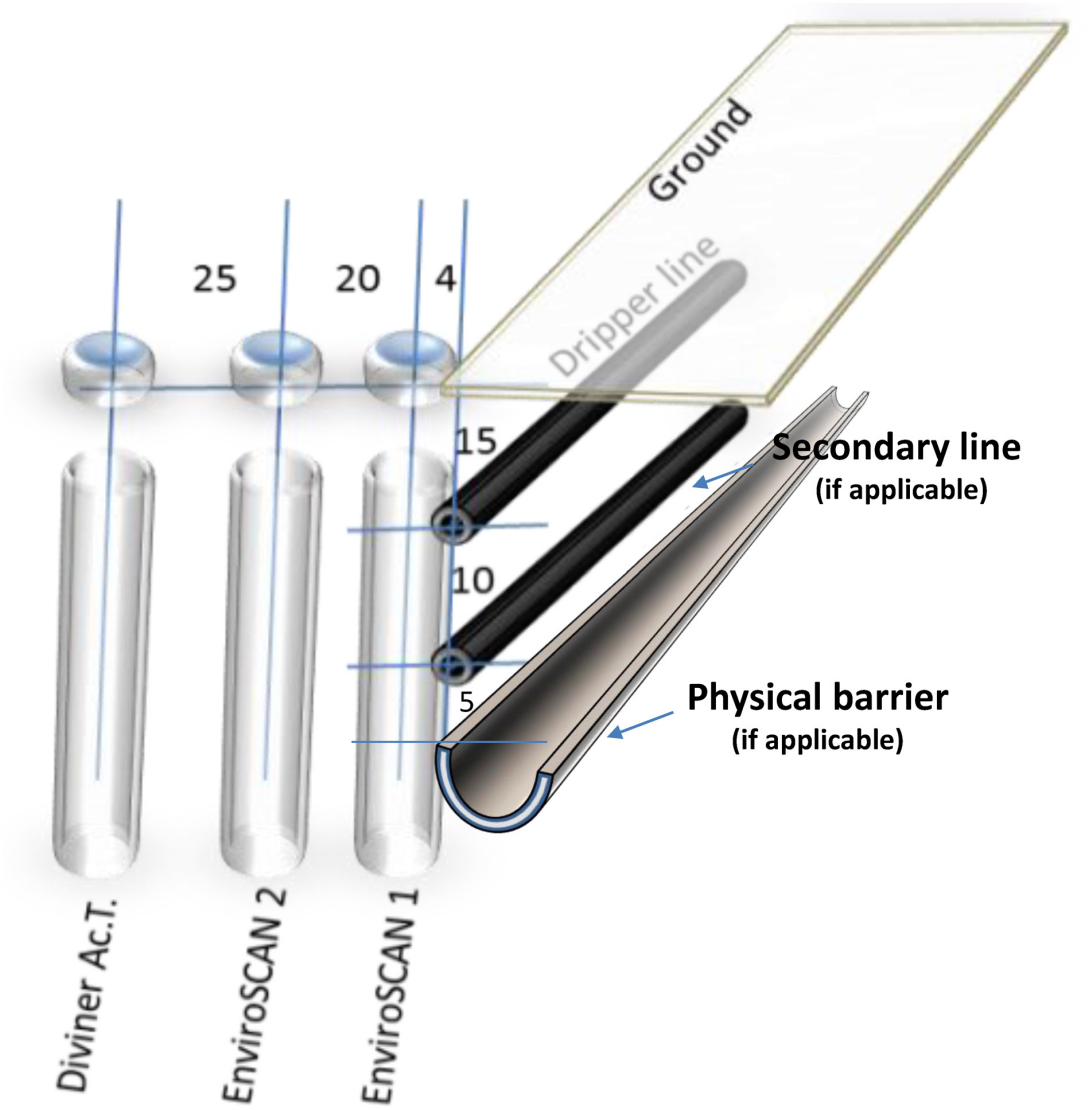
The access tubes locations and spacing.

To monitor water movement in the soil we installed capacitance probes, EnviroSCAN probes with 5 TriSCAN sensors each, Sentek technologies, Australia. The probes monitor water continuously, for each treatment we installed 2 probes; one of them bordering the emitters’ line, Fig 2, and the other is 20 cm apart (center to center), an additional access tube was installed 25 cm far from the second tube for on-demand measurements using another capacitance probe (Diviner 2000; Sentek technologies, Australia). Each probe consists of five sensors installed at 10, 20, 30, 40, and 60 cm depth. Data were collected manually every 3–5 weeks using a manual data logger. A rigor calibration process was performed according to the manufacturer’s manual as described in Elnesr et al. [34], readings were logged every 30 minutes through all the study period.

**Fig 3:**
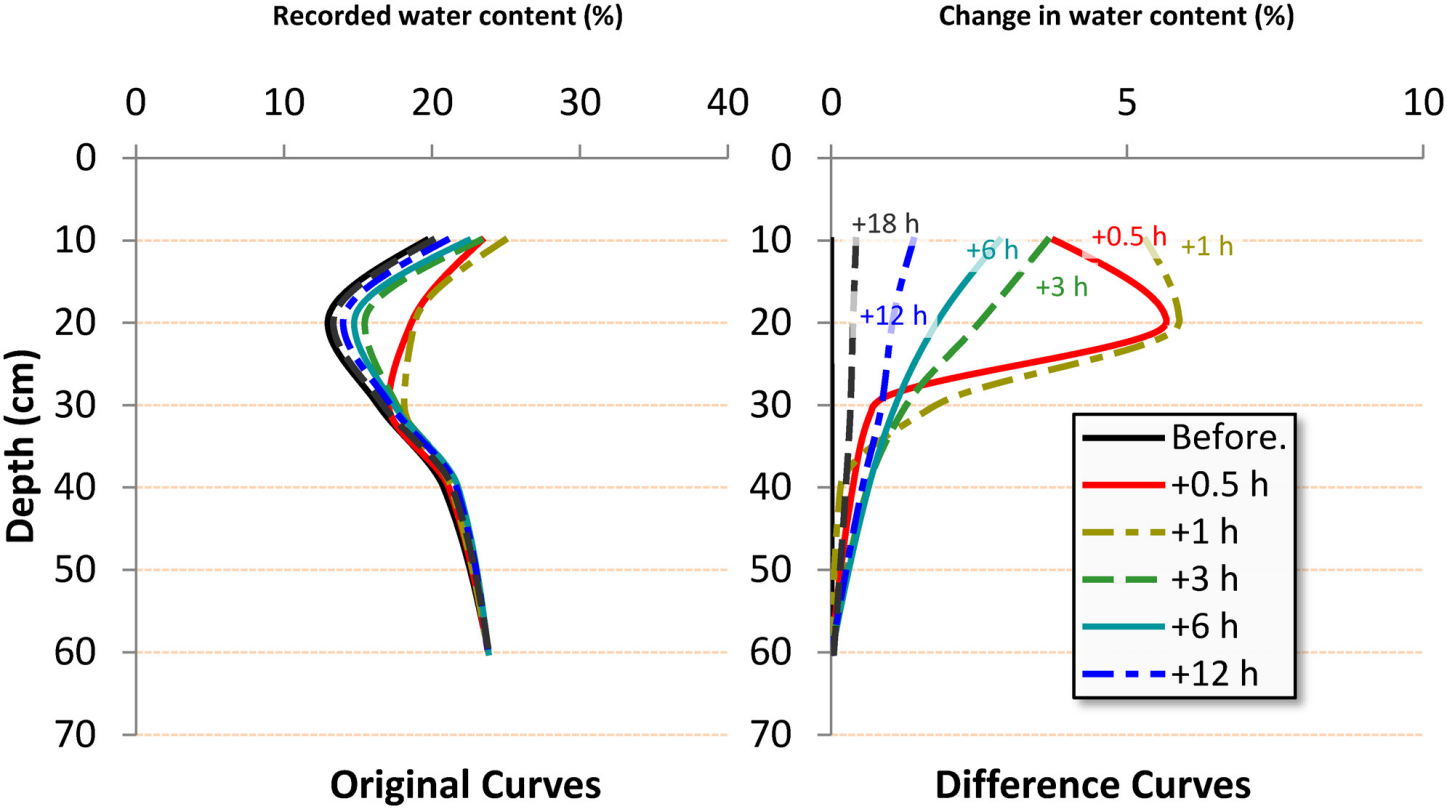
Converting water content charts to difference charts.

To limit the effect of soil heterogeneity, and to focus on the clear effect of the treatments, we calculated difference curves that show the change in water content from the initial time (before irrigation) to a specific time (0.5, 1, 3, 6, 12, 18 h after irrigation). These curves are easier to be explained than the original curves. Fig 3 shows a sample of both original and difference curves. The difference values (*WC*_*c*_) are simply calculated by subtracting the initial water content (*WC*_*i*_) values from the water content value of a specific time (*WC*_*t*_); e.g. if at *z* cm depth *WC*_*i*_ = 17.1%, *WC*_*t*_ = 19.8% then *WC*_*c*_ = 19.8–17.1 = 2.7% this was repeated for all the depths from 10 to 60 cm.

To qualify the irrigation process, we measured the irrigation water use efficiency (WUE, kg/m^3^), which is the ratio of crop yield to the applied irrigation water, Eq (2), [35].

$$WUE=\frac{1}{n}\sum_{r=1}^{n}\frac{Y_{r}}{Wa_{r}}$$ (2)

where *n*: number of replicates (rows) per treatment; *r*: counter; *Y*_*r*_: yield of the row (kg); *Wa*_*r*_: applied water to each row (m^3^/row).

Additionally, we calculated the water footprint of tomatoes (the amount of applied water per fruit, *FP*, L/fruit), which was introduced by Allan [36], Eq (3). The reference values of the FP for tomatoes and the methods of determination are obtained from Mekonnen and Hoekstra [37].

$$FP=\frac{1000}{n}\sum_{r=1}^{n}\frac{Wa_{r}}{FC_{r}}$$ (3)

where: *FC*_*r*_: fruit count per replicate.

### 2.7 Crop pre- and post-harvest measures

In the 8th week of tomatoes cultivation, we took one representative plant sample from each crop row to evaluate the growth indicators; We measured its length, counted its number of primary branches, and weighed each of its parts separately; leaves, stems and fruits (if any), and evaluated the water content in each of these parts. The water content evaluation was performed by taking about 100 g of each part (stem, leaves, fruit) then dried it at 70°C oven for 3–5 days (till no weight loss occur between two subsequent weighs); finally we calculated the water content percent as (Initial weight-dry weight)/dry weight*100. After about 80–90 days of cultivation the harvest process starts, while it is over after 4 to 5 harvests depending on the fruits maturity. For each harvest, each row of the 72 crop rows are weighed individually, the fruits of each row were counted, and then we selected 6 random fruits from each row’s yield. Portions of the fruits were sliced and dried at 70°C for 72 h to measure their dry matter and water contents. Other parts were homogenized using a fruit blender to determine the fruit chemical composition and fruit quality parameters. Total Soluble Solids (TSS, %) content were determined using a portable digital refractometer (PR-101, AT AGO, Japan). Titratable acidity, (TA, g 100 g^-1^ fresh weight), as citric acid was determined by potentiometric titration with 0.1 N NaOH against 9:1 dilution of fruit homogenate juice samples with distilled water. Vitamin C (mg 100 g^-1^ fresh weight, as ascorbic acid) content was measured by titration of homogenate fruit juice samples using 2,6 dichlorophenol- indophenol solution standardized in a solution of ascorbic acid with an identified concentration [38]. Finally, we measured the color of the fruits as an indicator of lycopene content [39]. Measurements of color were performed on 45 fruits per treatment using Hunter color-measuring instrument (Color Flex Hunter Lab, USA). The color space coordinates (L*, a*, b*) were directly read and stored by the instrument software. In this coordinate system, L* is a measure of lightness ranging from 0 (black) to 100 (white); a* positive values indicate amounts of red while negative values indicate amounts of green, and b*positive values indicate amounts of yellow while negative values indicate amounts of blue. It was reported that the chromatic index that correlates best with the lycopene content was (a*/b*)^2^, as the relationship is linear with r^2^ = 72–91% [40–42].

### 2.8 Cost analysis

The treatments that contain either the dual lateral or physical barrier techniques require additional equipment and installation costs than other treatments. Thus, we performed a cost analysis between the eight treatment combinations. The economic analysis is based on the methods of FAO [43], O’Brien et al [44], and Amosson et al [45]. The prices of the hardware and other expenses are based on the local market. The lateral line cost including drippers was 0.8$/m, and the manifold’s price was 1.5$/m. The fittings and other installation expenses was considered 30% of the total pipelines cost. The physical barrier price was 1.5$/m and the trenching and installation of the lateral lines was about 1.0$/m; increased 50% for the dual lateral installation, and 200% for the physical barrier installation. The system’s life was considered 8 years. The annual interest rate is 4%. The seasonal operation time is the sum of the daily operation time of each treatment according to the irrigation schedule (For the control treatment, the total time for seasons 1 and 2 were 40 and 34 h respectively). The electricity price was 0.08$/kWh. The pumps, tanks, flowmeters, and other seasonal labor, seeds, fertilizers, and land preparation expenses were computed from actual expenses in our field for the whole land and divided by 8 to get the expenses for each treatment. Each year includes two seasons, thus the annual rates are divided by two to calculate the seasonal rates. Finally, the selling cost of the yield was 0.5$/kg.

### 2.9 Statistical design

Data were subjected to analyses of variances (ANOVA) according to a factorial split-split plot design, with the intermittent application (S) as whole plots, dual drip (H) as the sub plots and the physical barrier (P) as the sub-sub plots. Means were tested with Fisher's least significant difference method (p<0.05). All the statistical analyses were accomplished using the Statistix package v10.0, Analytical Software. [46].

## Results

### 3.1 Crop results

According to the selected experimental design, the split-split plot design, we had the whole plot factor S, the sub-plot factor H, the interaction between them S×H, the sub-sub plot factor P, the dual interactions S×P, H×P, and the triple interaction S×H×P. The results are listed in Table 2. It is noticed from this table that most of the studied properties were significantly affected by the one or more of the treatments S, H, S×H, while there is no significant effect of the P treatment nor any of its combinations in most of the properties.

**Table 2:** Crop results and their statistical significance.

	**Yield weight (t/ha)**		**Fruits/plant**		**Fruit mass (g)**		**WUE (m^3^/kg)**		**Footprint (L/fruit)**		**Num. of primary branches**		**WC in leaves (%)**	
	Season 1	Season 2	Season 1	Season 2	Season 1	Season 2	Season 1	Season 2	Season 1	Season 2	Season 1	Season 2	Season 1	Season 2
S	***	ns	***	*	***	***	***	ns	***	ns	ns	ns	ns	**
0	49.8 a		60.4 a	49.3 a	102.4 b	134.2 b	16.6 a		6.6 b					83.6 b
1	39.1 b		46.5 b	42.5 b	111.0 a	157.4 a	13.6 b		8.5 a					84.6 a
H	***	***	***	**	ns	***	***	***	***	***	***	**	ns	***
0	35.7 b	46.2 b	42.6 b	42.2 b		137.1 b	11.9 b	12.0 b	9.0 a	11.7 a	4.0 b	5.2 b		83.5 b
1	53.3 a	57.7 a	64.4 a	49.6 a		154.4 a	18.3 a	17.4 a	6.1 b	9.1 b	5.5 a	6.1 a		84.7 a
S×H	ns	*	**	**	ns	ns	ns	ns	ns	ns	ns	ns	**	ns
0 0		44.0 b	38.1 d	41.8 b									85.7 a	
0 1		60.1 a	73.8 a	56.7 a									78.9 b	
1 0		48.5 b	55.0 b	42.6 b									80.3 b	
1 1		55.3 a	47.1 c	42.5 b									84.6 a	
P	ns	ns	ns	ns	ns	ns	ns	ns	ns	ns	ns	ns	ns	ns
S×P	ns	ns	ns	ns	ns	ns	ns	ns	ns	ns	ns	ns	ns	ns
H×P	ns	ns	ns	ns	ns	ns	ns	ns	ns	ns	ns	ns	ns	ns
S×H×P	ns	ns	ns	ns	ns	ns	ns	ns	ns	ns	ns	ns	ns	ns
	**Plant green mass (kg)**		**Plant height (cm)**		**Titratable acidity (%)**		**TSS (%)**		**Vitamin C (mg/100mL)**		**WC in fruits (%)**		**WC in stems (%)**	
	Season 1	Season 2	Season 1	Season 2	Season 1	Season 2	Season 1	Season 2	Season 1	Season 2	Season 1	Season 2	Season 1	Season 2
S	**	*	ns	***	*	ns	*	**	ns	**	*	ns	ns	**
0	0.97 a	1.19 b		76.5 b	18.8 b		4.81 b	4.74 b		0.436 b	94.8 b			81.5 b
1	0.76 b	1.42 a		87.5 a	20.4 a		5.04 a	4.99 a		0.457 a	95.1 a			83.2 a
H	***	***	***	***	ns	ns	ns	***	**	**	**	ns	***	***
0	0.55 b	1.00 b	59.4 b	75.5 b				5.06 a	0.381 b	0.428 b	94.7 b		82.4 b	80.5 b
1	1.18 a	1.60 a	75.4 a	88.5 a				4.67 b	0.440 a	0.465 a	95.2 a		87.0 a	84.2 a
S×H	ns	ns	ns	ns	ns	ns	ns	ns	ns	ns	ns	ns	ns	ns
P	ns	ns	ns	ns	ns	*	ns	ns	ns	ns	ns	ns	ns	*
0						20.7 b								82.9 a
1						21.8 a								81.8 b
S×P	ns	ns	ns	ns	ns	ns	ns	ns	ns	ns	ns	ns	ns	ns
H×P	ns	ns	ns	ns	ns	ns	ns	ns	ns	ns	ns	ns	ns	ns
S×H×P	ns	ns	ns	ns	ns	ns	ns	ns	ns	ns	ns	ns	ns	ns

**WUE**: Water Use Efficiency, **WC**: Water Content, **TSS**: Total Soluble Solids, **ns**: Not Significant at p = 0.05, **Asterisks** *, **, ***: significant up to p<0.05, p<0.01, p<0.001 respectively,

Means with the same letter are not significantly different from each other at the probability level above the numbers.

#### 3.1.1 Tomato yield

The total yield of tomato in season 1 was highly affected by S and H treatments (p<0.001), the dual lateral treatment (H_1_) yielded 53.3 t/ha, which is 49.5% more than the single lateral’s yield (H_0_ = 35.7 t/h), correspondingly, the application of S treatment caused 21.5% reduction in the yield (S_0_, S_1_ = 49.8 and 39.1 t/ha respectively). In the second season, the interaction of S×H was significant, and the highest yield was 60.1 t/h for the S_0_H_1_ treatment followed by the S_1_H_1_, S_1_H_0_, and S_0_H_0_ respectively, Table 2, where no significant difference between the two H_1_ means, nor between the H_0_ means. The fruits count per plant was affected by the S×H interaction in both seasons; the highest number of fruits per plant in the two seasons has resulted from the S_0_H_1_, with 73.6 and 56.7 fruits/plant respectively, the results of this treatment is significantly higher than the other treatment combinations, Table 2.

Unlike the previous measures that show negative effect of the S treatment, the fruit mass of the S_1_ treatments was higher than that of the S_0_ treatment for the two seasons, where S_1_ yielded 8.5 and 17.3% heavier fruits than S_0_ in season 1 and 2 respectively, Table 2. However, the effect of the H treatment was not significant in season 1, unlike season 2 that showed the positive effect of H_1_.

#### 3.1.2 Water use efficiency

The dual lateral technique, H, appears to have the sole effect on increasing the water use efficiency in the two seasons, Table 2, (54 and 45% increase in WUE when applying H_1_ in season 1 and 2 respectively). Conversely, the application of the intermittent irrigation, S, reduced the WUE in the first season by about 18%, while its effect was not significant in the second season. Similarly, the water footprint of the tomato was improved by applying H_1_ (e.g. the net applied water in season 1 was 9L/Fruit for H_0_, improved to be 6.1 L/fruit), and reduced by applying S_1_ (from 6.6 to 8.5 L/fruit for S_0_ and S_1_ in season 1 respectively).

#### 3.1.3 Vegetative growth

The overall growth of the plant appears to be affected by the H and S treatments as well, Table 2. The H technique increased the plant height from average of 59.4cm to 75.4cm and from 75.5cm to 88.5cm in seasons 1 and 2 respectively. However, the S application, had no significant effect on the plant height for the first season, while it had a positive effect on the second season. Likewise, the green mass was positively affected by H_1_ in the two seasons, while the effect of the S is negative in the first season, and positive in the second season. Furthermore, the results showed that the number of primary branches was positively affected by the H_1_ treatment and not affected by any other treatments.

The water content values in the stems, leaves, and fruits were somehow affected by the H and S treatments, Table 2. The water content is higher in the H_1_ than H_0_, and in S_1_ than S_0_ in the three measures. For the leaves, S×H interaction was significant in season 1 only, where the highest water content was the control (S_0_H_0_), with no significant difference from S_1_H_1_, then the S_1_H_0_ and the S_0_H_1_ treatments.

#### 3.1.4 Nutritional and maturity indicators

The analyses of the properties of the tomato juice showed that the titratable acidity was only increased by the S_1_ treatment in the first season, Table 2. Moreover, the TSS value was increased by S_1_ in both seasons, while decreased by H_1_ only in season 2. The maturity index (TSS/TA) was not significant for any of the measured treatments. Never the less, the content of vitamin C showed a significant increase by H_1_ in the two seasons and by S_1_ in the second season only. Finally, we found that no color indices were significantly changed by any of the studied treatments.

### 3.2 Changes in soil water content

**Fig 4:**
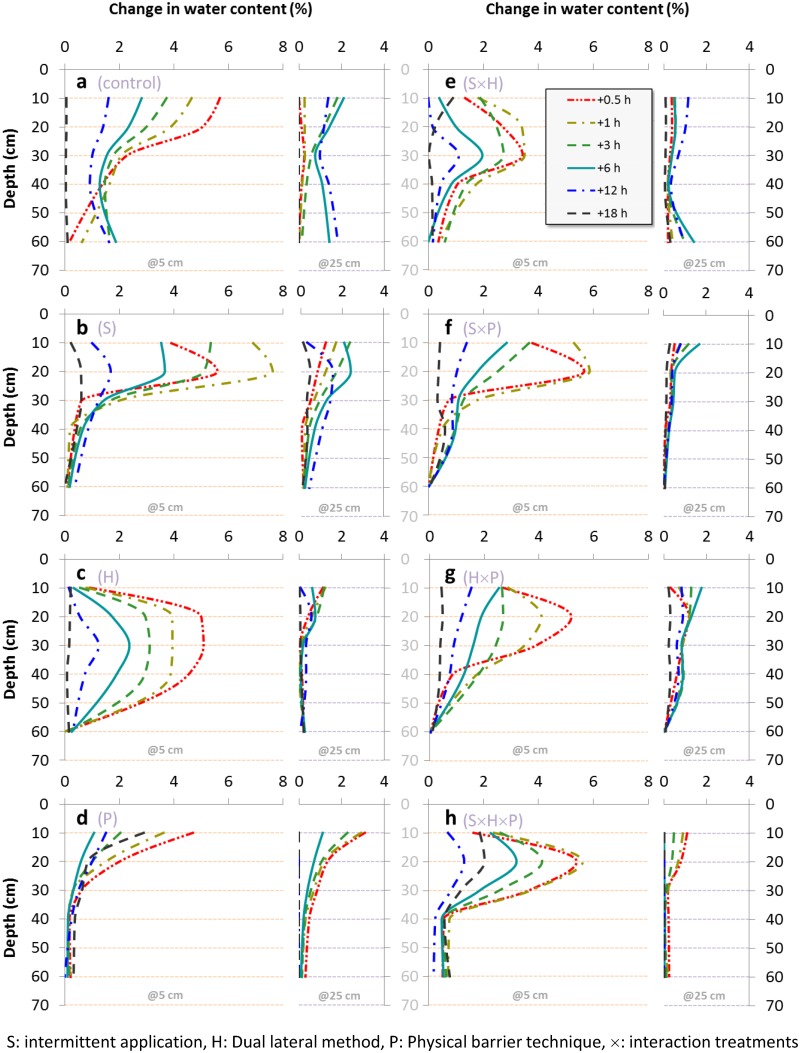
The change in soil water content at 5, 25 cm perpendicular distance from the buried emitters.

The curves that represent the change in water content (WC) are illustrated in Fig 4 for all the treatments (charts a to h). Each chart in the figure consists of two sets; the left set is the loggings of the probe located near the lateral line (@ 5 cm distance), while the right set of curves represents the distant probe, (@25 cm distance). The two sets help drawing a complete view of the water lateral, and vertical movement with time.

For all the charts in Fig 4, the right sets reflect a very small change in WC (less than 2%), this shows that the lateral movement of water is limited in all the treatments except for the physical barrier’s (Fig 4d) where the water approaches 3% increase at 10cm depth.

The control treatment (S_0_H_0_P_0_), Fig 4a, was the only treatment that shows gradual increase in WC up to 60cm depth which reflects deep percolation. The S treatments Fig 4b, 4e, 4f and 4h show that the maximum values of WC was reached after 1 hour, while the rest of the treatments the maximum water contents were reached sooner (after 0.5 h), this reflects the effect of pulsing.

In the P treatment Fig 4d and treatment combinations f-h we notice that water remains in the profile up to 18 hours after irrigation, for example, in chart d, at 10cm depth, WC approaches 3%, while it is almost zero in charts a, b, c, and e where the P is absent. This shows that the physical barrier keeps water in the root zone for a long time.

The effect of the H treatment is very clear in Fig 4c, as the two lateral lines unifies the WC vertically almost from 20 to 40 cm depth, this trend continued for each of the following logging times. It can be noticed too that the upward water movement is limited in the existence of the dual lateral (as the WC at 20 cm is much more than its value at 10 cm). On the other hand, we notice that the vertical shape of the WC curves has been reduced in the combined treatments charts (g and h), while it is slightly remains at the S×H treatment (e).

The increasing trend of WC near the soil surface appears only at the control and P treatments (a, and d), while the rest of the treatments show decreasing trend from10 to 20 cm depths. The increasing trend is not preferable as it increases the probability of water loss by evaporation.

### 3.3 The cost comparison

In the current experiment, each treatment occupies 1/8 of the total field area, which is 608/8 = 76 m^2^. We have conducted two cost analysis studies, one for the current study’s area, and the other was simulated for a four hectares square plot (200 x 200 m^2^) with the same conditions. The summary of the cost analysis of the current study’s area is shown in Table 3, while the details of both the current area and the simulated area are attached in S1 and S2 Tables.

**Table 3:** Cost analysis of the studied systems.

	S_0_H_0_P_0_	S_0_H_0_P_1_	S_0_H_1_P_0_	S_0_H_1_P_1_	S_1_H_0_P_0_	S_1_H_0_P_1_	S_1_H_1_P_0_	S_1_H_1_P_1_
**Fixed costs**								
Dripper lines ($)	72	72	144	144	72	72	144	144
Manifold ($)	12	12	15.6	15.6	12	12	15.6	15.6
Fittings ($)	3.6	3.6	4.7	4.7	3.6	3.6	4.7	4.7
Installation ($)	90	270	135	270	90	270	135	270
Physical Barrier ($)	0	135	0	135	0	135	0	135
Sum of network costs	178	493	299	569	178	493	299	569
Other costs (pumps, etc…)	800	800	800	800	800	800	800	800
**Total network costs**	**978**	**1293**	**1099**	**1369**	**978**	**1293**	**1099**	**1369**
Annual depreciation	122	162	137	171	122	162	137	171
Yearly interest value	39	52	44	55	39	52	44	55
Total annual costs	**161**	**213**	**181**	**226**	**161**	**213**	**181**	**226**
**A- Total Fixed Costs/season**	**81**	**107**	**91**	**113**	**81**	**107**	**91**	**113**
**Variable Costs**								
Operation time/season	40	40	20	20	40	40	20	20
Required power	1.5	1.5	2.25	2.25	1.5	1.5	2.25	2.25
Required energy	60	60	45	45	60	60	45	45
Electricity Price/kWh	4.8	4.8	3.6	3.6	4.8	4.8	3.6	3.6
Plant protection.	25	25	25	25	25	25	25	25
Fertilizers	13	13	13	13	13	13	13	13
Labor	83	83	83	83	83	83	83	83
Seeds	2	2	2	2	2	2	2	2
Preparation	20	20	20	20	20	20	20	20
**B- Total Variable Costs/season**	**148**	**148**	**147**	**147**	**148**	**148**	**147**	**147**
**C- Total seasonal costs (A+B)**	**229**	**255**	**238**	**260**	**229**	**255**	**238**	**260**
*Returns*								
Yield (kg)	648	624	909	916	615	600	779	771
**D- Income (as 0.5$/kg)**	**324**	**312**	**454**	**458**	**308**	**300**	**390**	**385**
**E- Profit (D-C)**	**95**	**57**	**216**	**198**	**79**	**45**	**152**	**125**
**F- Profit margin (E/C)**	**41%**	**22%**	**91%**	**76%**	**34%**	**18%**	**64%**	**48%**

The cost analysis in Table 3 showed that the highest profit margin was obtained when using the dual lateral (H) without either P or S (S_0_H_1_P_0_, = 91%) followed by the treatment of H with P only (S_0_H_1_P_1_ = 76%) then H with S (S_1_H_1_P_0_; 64%). On the other hand, the least margin was obtained from S_1_H_0_P_1_treatment, which combines S and P without H (18%). Although the fixed costs of the H treatment are doubled at the dripper lines’ item, but the higher yield of the treatment led to more than double of the profit margin comparing to the control treatment (S_0_H_0_P_0_). Additionally, the small operation time of the H treatment reduced the pumping costs as well. The simulated area of 4 ha showed similar results, S2 Table, as the highest profit margin was 135% for S_0_H_1_P_0_, followed by that of S_0_H_1_P_1_, 120%, and the least value of the profit margin was 47% for S_1_H_0_P_1_.

## Discussion

The physical barrier in our study showed non-significant effects over all the studied properties. These results may be due to one or more of the following conditions: the soil texture, the barrier’s layout (width, shape, and depth), or the crop type. The main reason for using the physical barrier is to limit the downward water movement due to high soil permeability [8,10]. As a result, the physical barrier produced higher yields (especially for tomatoes) when applied to sandy textured soils as reported by some investigators [11,12]. Accordingly, since our soil’s texture is sandy loam with moderate permeability that it does not have the problem of deep percolation especially with low-flow emission like drip irrigation, then the barrier had no effect on the current soil texture. The barrier’s shape, width and depth may also have influence the barrier’s performance. Our barrier’s was narrow (11 cm) if compared to Ismail et al. [14], 50 cm, but it is wider than that of Brown et al. [10], 8 cm, both of them reported good results of their barriers, hence, it can be concluded that the width was not the reason of our results. Furthermore, the depth of our barrier, 30 cm, was similar to that of the previous works with positive results, e. g. Ismail et al. [14]. With similar concept to the physical barrier, the capillary barrier’s effect varies by the crop type, [47] as they reported good results for pepper and lettuce and negligible effects on tomatoes and melons, however, tomato crop was positively affected by the physical barrier in sandy soil [14]. Thus, the negative result of the physical barrier in this study appears to be affected only by the soil texture. Additionally, it is important to bear in mind that physical barriers may increase water loss by evaporation by forcing water to move upward as shown in the current study. Nevertheless, physical barriers have some harmful effects on plat roots and may cause accumulation of unwanted chemicals in the root zone [13], thus it should be avoided unless really necessary. In conclusion, it is only advised to apply this technique for light textured soils when the water is excessively lost by deep percolation.

The results showed that the intermittent drip had a negative effect on the yield quantity and the water use efficiency, while it had a positive effect on the fruit size (mass) and on the height of the plant in the second season. This means that applying the irrigation water all-at-once is better to gain higher yields, but with smaller fruits. The intermittent application showed a significant increase in the maturity measures; TSS, and titratable acidity, this improvement in fruit quality combined with the significant increase in the fruit mass is a good indicator of quality improvement. Thus, in cases that the fruit size and ripening signs are marketing aspects, the intermittent application should be considered.

On the other hand, we should bear in mind that the intermittent application results are affected by the pulse rate, the soil texture and water content, and the type and age of the crop [20,48,49]. This urged some investigators to believe that the future of this technique is not so promising, as it was tested with many frequencies, many crops, and in many soil-textures, and all the results was not encouraging (Alon Ben-Gal, personal communication, November 6 2014). Correspondingly, the good results of the intermittent application in some published research may be attributed to limiting deep percolation [23], or limiting emitters clogging [16], these two reasons may be the reason of increasing yields and WUE. Consequently, if the intermittent application is applied in some systems that do not suffer of deep percolation and emitters clogging problems it might not enhance the growing conditions. Another factor that should be considered, that we have noticed that each time the valves close, the emitters leak some excess water due to the effect of the air relief valve. Hence, the more valve closing events, the more water is wasted. Since the S_1_ treatment requires three valve closes instead of one for S_0_, then more water is wasted in the S_1_ treatments. This may explains the increase of water discharged to the S_1_ plots, and hence causes the reported decrease of water use efficiency. In conclusion, the intermittent application could be considered only if the soil texture causes deep percolation, or the system is exposed to potential emitter clogging.

The dual lateral method showed positive results in almost all the studied indicators. It produced greater yields and plant growth, better WUE, higher values of vitamin C, while it had positive effect on the fruit mass and the TSS in the second season only and no effect on the titratable acidity, in addition to its high profit margin over all the studied combinations. These results showed that this method has a very good effect on yield’s quantity (in the two seasons), quality (in the cold season), and economically. The good results of the dual lateral agrees with Ismail et al.[14] when the vertical distance between the two laterals was 10cm, while they reported that if this distance is more, then the dual lateral produced less yield as the water of the secondary lateral escaped out of the reach of the rootzone. This lead to a conclusion that the effect of this technology is related to water distribution along the root system, which is reflected on the water distribution patterns, Fig 4c. In similar setup, Zotarelli et al. [50] placed two lateral lines, one at 15 cm below soil surface, and one at the soil surface (for fertigation), they found that this placement increased the root length distribution 48–54% than surface drip irrigation. Although the dual lateral method involves two subsurface laterals, but their results support our assumption that the placement of two lateral lines one above each other enhances the water distribution patterns leading to higher quantity and quality yields. According to the soil-water distribution patterns, the placement of two lateral lines instead of one leads the water vertical distribution to be uniform up to 40 cm, Fig 4c. this distribution water pattern may be the main reason of this good results of the dual lateral method. However, the effect of the dual lateral on the root distribution needs more research to confirm this hypothesis.

As the results showed that none of the treatments had a significant effect on any of the tomato skin color indices, and since the lycopene content is directly proportional to the (a*+b*)^2^ color index [40–42], we can conclude that the lycopene content is not affected by any treatment as well. This agrees with the studies that reported that Lycopene is affected by water deficiency [51,52], as in this study all the treatments were subjected to the same amount of water and no water deficiency. This shows that the positive effects of the dual lateral and intermittent techniques are due to the application method not to any difference in the application quantity. This proves that these two techniques are capable of enhancing yield quantity and quality using the same amount of water.

## Summary and Recommendations

Each of the three tested techniques has different effect on the wetting pattern and the crop growth. The major shift in crop yield was caused by the dual lateral technique, where it appears to enhance the wetting pattern; this hypothesis, however, requires more research. The dual lateral technique is recommended for its good yield results especially that it is the most economical treatment above all others despite the increase of its fixed costs. The intermittent application improved the fruits size, ripening degree, and content in vitamin C, while it caused reduction in the total yield of the crop, however, it is more economic than the control treatment. The previous studies showed that the intermittent application limits deep percolation and emitters’ clogging, while in this study due to our conditions, we have neither deep percolation nor emitters clogging, and hence the effect of this technique does not appear in this study. The intermittent application improved some of the tomato quality measures, and it is recommended to consider it when the quality is more important than quantity especially on sandy soil. The physical barrier had no effect on any of the important measures, while it appears to increase the lateral and upward movement of water, where it can increase evaporation and reduce water use efficiency, additionally, it is not economic in the current study’s conditions. The previous studies who reported positive effect of this technique were of sandy textured soils with high permeability, combining this with the other studies that warn of the physical barrier hazards like causing chemical toxicity or growth inhibition of roots, we can recommend to avoid using this technique unless really needed in extremely high permeable soils.

## Supporting Information

S1 Table A comparative cost analysis of eight treatments, each of them cultivated in 76 square meters. (XLSX)

S2 Table A comparative cost analysis of eight treatments, each of them cultivated in 40,000 square meters. (XLSX)
